# Co-Prevalence of Virulence and Pathogenic Potential in Multiple Antibiotic Resistant *Aeromonas* spp. from Diseased Fishes with *In Silico* Insight on the Virulent Protein Network

**DOI:** 10.3390/life12121979

**Published:** 2022-11-25

**Authors:** Nabanita Chakraborty, Basanta Kumar Das, Asit Kumar Bera, Simanku Borah, Debasmita Mohanty, Anil Kumar Yadav, Jeetendra Kumar, Satish Kumar Koushlesh, Thangjam Nirupada Chanu, Soumya Prasad Panda, Ravali Vallangi

**Affiliations:** 1 Regional Centre, Central Inland Fisheries Research Institute (ICAR), Guwahati 781006, India; 2 Central Inland Fisheries Research Institute (ICAR), Barrackpore 700120, India; 3 Regional Centre, Central Inland Fisheries Research Institute (ICAR), Prayagraj 211002, India

**Keywords:** *Aeromonas*, Assam wetland fisheries, virulence gene, antibiotic resistance, pathogenesis, protein–protein interaction

## Abstract

*Aeromonas* species exhibit widespread presence in food, poultry, and aquaculture. They are major multi-drug-resistant fish pathogens. This study aims to identify *Aeromonas* species harbouring virulence genes aerolysin, flagellin, and lipase from diseased fishes of Assam wetlands with association with antibiotic resistance and in vivo pathogenicity. One hundred and thirty-four *Aeromonas* strains were isolated and thirty representative species identified using genus-specific 16S rRNA gene amplification. *A. veronii* was most prevalent (53.7%) followed by *A. hydrophila* (40.2%), *A. caviae* (4.47%), and *A. dhakensis* (1.49%). Ninety percent (90%) of strains harboured at least one of the studied virulence genes: *aer*A (73.3%), *lip* (46.6%), and *fla*A (26.6%). The highest multiple antibiotic resistance (MAR) index 0.8 corresponded to *A. hydrophila* DBTNE1 (MZ723069), containing all the studied genes. The lowest LD50 values (1.6 × 106 CFU/fish) corresponded to isolates having both *aer*A and *lip*. β-lactams showed utmost resistance and lowest for aminoglycosides. There was a significant (*p* < 0.05) Pearson chi-square test of association between the occurrence of virulence and antibiotic resistance. The in silico protein–protein interaction revealed important drug targets, such as σ28 transcription factor, aminoacyl-tRNA synthetase, and diacylglycerol kinase, with significant (*p* < 0.05) enrichment. This study suggests that fish-isolate *Aeromonas* strains represent potential threat to aquaculture with subsequent risk of transferring antibiotic resistance to human pathogens.

## 1. Introduction

Assam (26.2006° N, 92.9376° E) is a major fish-producing and -consuming state in northeast India with 3.06 lakh metric tons of fish production [1]. Flood-plain wetlands (1197 no.; 100,815 ha) in the form of shallow depressions, such as river basins, oxbow lakes, or floodplain wetlands, are one of the major fisheries resources of the state [2]. These resources have a high potential productivity of 2000 kg/ha/year [3]. Floodplain wetlands of the state managed solely through capture fisheries has a yield ranging from 156.6–206.4 kg/ha/year [4], while those managed through culture-based fisheries have a high average production of 704.60 kg/ha/year [5]. These wetlands are dominated by small indigenous and carnivorous fishes, including *Amblypharyngodon mola*, *Puntius* spp., *Bangana dero*, *Colisa* spp., *Chanda* spp., *Mystus vittatus*, *Ambassis* spp., *Mastacembelus armatus*, *Labeo rohita*, *L. catla*, *L. bata*, *Ompok bimaculatus*, *Channa punctatus*, *Clarias batrachus*, *Wallgo attu*, small prawns, etc. [6,7,8,9]. However, fish diseases have emerged as a threat in the state fisheries and aquaculture industry. The very high fish species diversity, aquaculture intensification of multiple species, and subtropical humid climate of the state have cumulatively favoured a greater number of fish pathogens during almost all seasons [10]. Most commonly encountered health ailments of fishes in Assam include epizootic ulcerative syndrome, hemorrhagic septicemia, abdominal dropsy, fin and tail rot, and Popeye deformity [11], caused mainly by *Aeromonas* spp., *Pseudomonas* spp., *Flavobacterium* spp., and *Edwardsiella* spp. [12]. Motile *Aeromonas* septicemia (MAS) and Motile *Aeromonas* Infection [13] are the most severe, fatal bacterial diseases caused by motile *Aeromonas* species, namely, *A. hydrophila*, *A. sobria*, and *A. caviae*. These are opportunistic Gram-negative facultative anaerobes with a high degree of habitat and temperature adaptability [14]. They can colonize and multiply in freshwater (chlorinated and non-chlorinated), brackish, and sewage water. The interaction of fishes with the aeromonads becomes deadly under deteriorated water quality conditions, which compromises fish immunity and makes them vulnerable to infection [15].

The pathogenesis of *Aeromonas* spp. is quick and intense due to the presence of multiple virulence factors, including the production of toxins (cytotoxins, hemolysins, and enterotoxins), the ability of adhesion (adhesins), ability to locate toward the favourable environment (flagellin), and hydrolytic enzymes (lipase and protease) [16,17]. Heat-labile cytotonic enterotoxin of *A. hydrophila* reportedly causes diarrhea in humans and animals. They also cause human infections, such as gastroenteritis or skin soft-tissue infections, and are considered human pathogens [18,19]. Aerolysin, a Type II pore-forming exotoxin (PFT), is the most important virulence factor of *Aeromonas* spp., and it is widely distributed across species [20]. The three most important species, *A. hydrophila*, *A. caviae*, and *A. veronii*, are reportedly responsible for more than 85% of the human infections caused by this genus [21]. The presence of these virulence factors not only enables the bacteria to invade their host’s defence mechanisms but also to overcome antibiotic and antimicrobial challenges by increasing the minimum inhibitory concentration [22]. Exotoxins destroy the intermediate products of the antibiotic activity pathway and modify the pathway enzymes [23]. Genus *Aeromonas* is considered naturally resistant to β-lactam antibiotics. The β-lactamase enzyme secreted by the bacteria cleaves the β-lactam ring and confers resistance against all β-lactam antibiotics [24,25].

Antibiogram information of a pathogen is vital for the management of the diseases caused by the given pathogen. Conventional antibiotics, commonly used in aquaculture practices, have been deliberated focusing on diseases in human and terrestrial animals [26]. Class and dose of antibiotics used in fish disease treatment and their application method, i.e., oral or feed supplementation, have not yet been quantitatively validated under standard drug approval procedures for most fish species. Lack of this information results in indiscriminate use of antibiotics by fish farmers. A well-documented consequence is the development and transmission of antibiotic resistance recorded in *Aeromonas* spp., against several antibiotics [27]. There are earlier studies which reported the multidrug resistance of *Aeromonas* strains isolated from carps [25]. Similar results were obtained for β-lactam antibiotics [28]. A comparative study revealed that 33.3% of the *Aeromonas* strains from farmed chicken were resistant to tetracycline, and 31.6% strains of water origin exhibited resistance against Amoxicillin–clavulanic acid [29].

Multidrug-resistant motile *Aeromonas* spp. Can easily transmit themselves or their resistance gene via drinking water, aquaculture products, or clinically significant human pathogens [30]. Sulphonamide-resistant *Aeromonas salmonicida* was reported in USA during 1955. Years later, multidrug-resistant *A. salmonicida* strains were isolated from Japan [31]. Resistance gene transmission in *Aeromonas* accounts for the presence of genetic jumping elements including class 1 integrons, transposons, and genomic islands [32,33] which are also considered as major virulence determinants of bacteria [34]. Therefore, we are rapidly approaching a time where few drugs would remain for treating microbial diseases. A vivid knowledge about these virulence factors and their associated protein networks involved in their regulatory pathways can help in identifying effective drug target resources.

In the present study, we investigated the phylogenetic origin of isolated *Aeromonas* strains from the diseased fishes of Assam wetlands and their association with virulence genes with antibiotic resistance patterns.

## 2. Materials and Methods

### 2.1. Sample Collection

**Figure 1:**
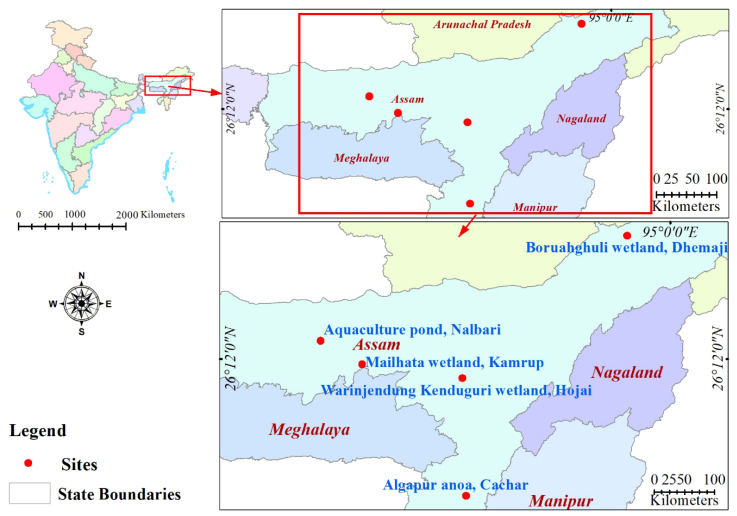
Study area map of different sampling sites in Assam, India.

Sample collection was conducted from 134 moribund fishes mostly belonging to eight different species: *Systomus sarana*, *Oreochromis niloticus*, *Pangasianodon hypophthalmus*, *Sperata aor*, *Labeo catla*, *Labeo bata*, *Cirrhinus mrigala*, and *Channa marulius*. Fishes with swollen and protruding eyes and visible hemorrhagic lesions on the skin and pectoral fins were used for bacterial isolation. Samples were collected during the period April–June 2020, from five different locations: Mailhata wetland, Kamrup; Warinjendung Kenduguri wetland, Hojai; aquaculture pond, Nalbari; Algapur anoa, Cachar; and Boruahghuli wet-land, Dhemaji (Figure 1). Fishes were terminally anesthetized using clove oil (200 μL/L) (Merck, Darmstadt, Germany), then washed thoroughly with sterile phosphate-buffered saline (PBS, pH 7.2), and finally, their body surface was disinfected using 70% ethyl alcohol. Blood samples were drawn through the caudal venipuncture and transferred in Tryptic Soya Agar (TSA, Hi-media) plates. Plates were then incubated, at 37 °C, for 24 h. Single colony isolates were sub-cultured thrice on fresh TSA plates to obtain a pure culture. All the isolates were stored in Luria-Bertani broth containing 20% glycerol at −20°C until further use.

### 2.2. Bacterial Isolation and Identification

Isolates were preliminarily screened for *Aeromonas* by growing in alkaline peptone water enriched with ampicillin (10 μg/mL) (Hi-Media, Maharashtra, India), ampicillin dextrin agar supplemented with ampicillin (10 μg/mL), and vancomycin hydrochloride (2 μg/mL) [15].

Identification of the strains was confirmed by twenty-five biochemical tests viz., ONPG (β-galactosidase), urease, lysine utilization, nitrate reduction, ornithine utilization, malonate utilization, Voges Proskauer’s (VP), esculin hydrolysis, phenylalanine deamination, citrate utilization, H_2_S production, indole, methyl red, oxidase, and the utilization of arabinose, adonitol, xylose, rhamnose, melibiose, cellobiose, saccharose, raffinose, trehalose, glucose, and lactose [35].

Genomic DNA of the bacterial isolates was extracted using the QIAamp DNA mini kit (Qiagen, Valencia, CA, USA). *Aeromonas* genus-specific 16S rRNA gene amplification and sequencing was done using a standard protocol [36]. A combined phylogenetic tree was constructed through the neighbour-joining method using the MEGA X program (Molecular Evolutionary Genetics Analysis, State College, PA, USA) [37]. Percentage of replicates was shown in the related taxa grouped in the bootstrap analysis (1000 replicates) next to the branches [38].

### 2.3. Antibiotic Susceptibility Testing

Susceptibility test to antimicrobials was performed using the Kirby–Bauer disk diffusion method according to the standard protocol M45 of the Clinical and Laboratory Standards Institute (CLSI) [39]. Twenty-five antibiotics of ten different classes were used for the assay. Single colonies from each bacterial isolate were dissolved in 0.85% saline solution to reach 0.5 McFarland standards (~1.5 × 10^8^ CFU/mL). Following the spread of the bacterial suspension on Muller Hilton Agar plates with a sterile cotton swab, the antibiotic disks were placed onto the media using a disk dispenser and incubated at 37 °C for 16–18 h. Analysis was performed in triplicates. *A. hydrophila* ATCC 7966 was used as a positive control.

The multiple antibiotic resistance (MAR) index was calculated following the formula given by Nandi and Mandal [40];

MAR = Number of antibiotics to which isolates are resistant ÷ Number of total antibiotics exposed

Results were interpreted according to CLSI M45 and those not mentioned in the CLSI were referred from other studies [41]. Isolates with MAR index values of ≤0.2 were considered low risk, and those with exceeding 0.2 were registered as high risk.

### 2.4. Virulence Gene Detection by PCR

Amplification of the aerolysin (*aer*A), lipase (*lip*), and the structural gene flagellin (*fla*A) were performed using the template DNA extracted from the confirmed *Aeromonas* strains. Primers for amplification were obtained from published studies with minor modifications (Table S1). PCR was standardized by altering the annealing temperature and other thermal cycling conditions. Amplification with a reaction volume of 25 μL was carried out using the Qiagen PCR master mix (QIAGEN, Hilden, Germany). Cycling conditions consisted of initial denaturation at 94 °C for 3 min followed by 35 cycles of denaturation at 94 °C for 30 s, annealing for 45 secs at 1 °C below the mean T_m_ of the primers, and extension at 72 °C for 2 min. Amplicons were visualized in 1.5% agarose gel run in 1% Tris-acetate-EDTA (TAE) buffer using a 100-bp DNA ladder as molecular marker. Representative samples were sequenced for each gene.

### 2.5. LD50 Determination

The LD50 values were calculated as a measurement of virulence. Three isolates each containing each of the virulence genes and their combinations (*n* = 21 for 7 groups) were used to challenge healthy and well acclimatized tilapia fish (*Oreochromis niloticus*) with average body weight 12 ± 1.02 gm. For the purpose of calculating the lethal dose (LD50) for each *Aeromonas* isolate, and evaluating the cumulative mortality, the experimental pathogenicity test was performed. Tryptic Soy Broth (TSB) and Tryptic Soy Agar (TSA) were used to culture the 21 *Aeromonas* isolates for 24 h at 37 °C. Concentrations ranging from 1.1 × 10^10^ mL^−1^ to 6.6 × 10^10^ mL^−1^ of the pure cell pellets were diluted in sterile phosphate-buffered saline (PBS). The experimental fish were injected intraperitoneally with bacterial suspension with six increasing dilution in ten folds with an injected volume of 200 μL/fish. A control group injected with PBS was maintained. The LD50 was calculated using Reed and Muench method.

### 2.6. In Silico Protein–Protein Interaction Network Analysis of Virulence Genes

Biological interaction of the *aer*A, *lip*, and *fla*A genes and proteins could be vividly explained by analysing their network and pathways. In this study, two web-based network tools were used to investigate these interactions. The Search Tool for the Retrieval of Interacting Genes/Proteins (STRING), version 11.0 [42], was used to visualize the protein–protein interactions (PPI) based on gene neighbourhood, fusion, co-expression, co-occurrence, and homology. It works on phylogeny-based pre-computed networks. Full network analysis (medium confidence; 0.400) was performed considering the physical protein associations quantified by the string db score. In the networks, nodes and edges represent the proteins and interactions, respectively. Cytoscape version 3.8.2 software was used to visualize the PPI network [43,44]. Target gene *aer*A was selected from *A. veronii* strain B565. The *lip* and flagellin *fla*A target genes were selected from *A. hydrophila* ATCC 7966. PPI enrichment value was used as a qualitative parameter for biological network validity evaluation. Statistical analysis was performed using SPSS 20 (IBM SPSS, Chicago, IL, USA). The Chi-square test was used to evaluate the association between the virulence factors and the MAR index. Strength of the association was studied by the Goodman and Kruskal tau directional measures and Phi symmetric measures. *p*-value of *p* < 0.05 was considered statistically significant.

## 3. Results

### 3.1. Aeromonas Isolation and Identification with Phylogenetic Studies

**Figure 2:**
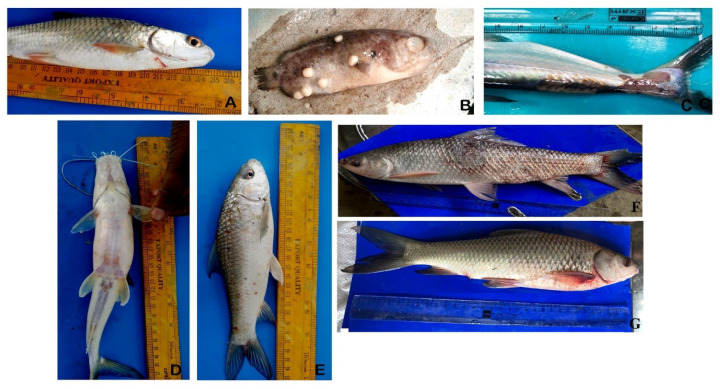
Disease caused by *Aeromonas* spp. in (**A**) *Systomus sarana* (**B**) *Oreochromis niloticus* (**C**) *Pangasianodon hypophthalmus* (**D**) *Sperata aor* (**E**–**G**) *L. catla* indicating major clinical signs.

A total of 197 bacterial isolates were recovered from diseased fishes (Figure 2). Out of them, 134 isolates were confirmed to belong to *Aeromonas* spp. As such, multiple bacterial isolates were obtained from a single moribund fish. Isolates were grown in alkaline peptone water containing ampicillin (10 μg/mL) (Hi-Media, India) and produced bright yellow colonies on ampicillin dextrin agar (US Environmental Protection Agency, 2001) (Figure S1). All isolates were positive for ONPG, lysine utilization, nitrate reduction, phenylalanine deamination, citrate utilization, indole, oxidase, VP, esculin hydrolases, saccharose, trehalose, and glucose utilization. *A. veronii* was positive for ornithine utilization, cellobiose, and raffinose. Isolates were negative for methyl red, malonate utilization, adonitol, rhamnose, and xylose (Table 1).

Strains were further confirmed by PCR amplification of a 953-bp 16SrRNA gene fragment using genus-specific primer (Table S1) sequencing (Figure S3A). *A. hydrophila* ATCC 7966 was kept as standard positive control throughout the experiment. At a species level, 53.7% of the *Aeromonas* strains were identified as *A. veronii*, 40.2% as *A. hydrophila*, 4.47% as *A. caviae*, and 1.49% as *A. dhakensis*. Thirty representative *Aeromonas* genus-specific sequences were submitted to NCBI.

**Figure 3:**
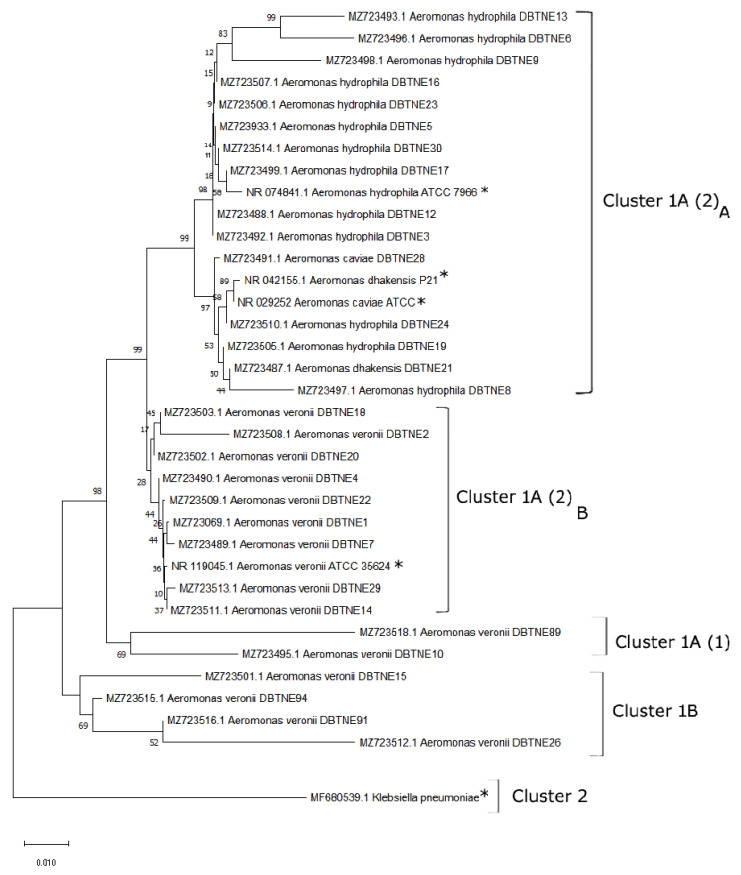
Phylogenetic tree analysis of *Aeromonas* sp. based on 16S rRNA gene sequences following Neighbor-Joining method by the MEGA X software. The numbers next to the branches indicate percentage values for 1000 bootstrap replicates. Bootstrap values above 50% are shown at the nodes. The reference strains taken from NCBI are indicated by an asterisk.

Maximum likelihood algorithm was applied to construct the phylogenetic tree, which showed evolutionary relationship among bacterial strains. The tree was divided into two major clusters. Cluster 1 contained all nucleotide sequences considered in this study along with references marked with an asterisk. Cluster 2 contained an out-group sequence *Klebsiella pneumoniae* strain during this study. Fifteen *A. veronii* strains were grouped into Clusters 1A (1) and (2)_B_, sharing 98% homology. Fourteen *A. hydrophila* strains and one of both *A. dhakensis* and *A. caviae* strain were grouped into Clusters 1A and (2)_A_ along with reference strains from NCBI (Figure 3). Clusters 1A (2)_A_ and (2)_B_ share 99% homology, while no homology was observed between Clusters 1 and 2.

### 3.2. Prevalence of Virulence Genes

Prevalence of virulence genes in the *Aeromonas* spp. was variable across the species. Overall, 90% of the strains contained the studied virulence genes. PCR amplification of a 431-bp fragment revealed that approximately 76.6% of the *Aeromonas* strains were carrying the *aer*A gene (Figure S3B). At the species level, 52.17%, 34.78%, 4.35%, and 4.35% of *A. veronii*, *A. hydrophila*, *A. caviae*, and *A. dhakensis* carried the gene, respectively. Two representative *aer*A gene from the *Aeromonas* isolates (*A. dhakensis*; strain DBTNE21: MZ596742, and *A. veronii*; strain DBTNE22: MZ564197) were sequenced and submitted to NCBI.

Approximately 46.6% of the *Aeromonas* strains were carrying the *lip* gene, identified by the 247-bp PCR amplicon [Figure S3C]. Prevalence of the *lip* gene was the highest in *A. hydrophila* (50%) followed by *A. veronii* (35.7%), *A. caviae* (7.14 %), and *A. dhakensis* (7.14%). One representative *lip* gene from the isolates (*A. caviae*; strain DBTNE28: MZ596741) was sequenced and submitted to NCBI.

Approximately 26.6% of *Aeromonas* strains were carrying the *fla*A gene identified by the 608-bp PCR amplicon (Figure S3D). One representative *fla*A gene from the isolates (*A. veronii*; strain DBTNE4: MZ596744) was sequenced and submitted to NCBI. At the species level, 50% of *A*. *veronii*, 37.5% of *A. hydrophila*, and 12.5% of *A. dhakensis* carried the gene.

### 3.3. Antibiogram of the Confirmed Aeromonas Strains

**Figure 4:**
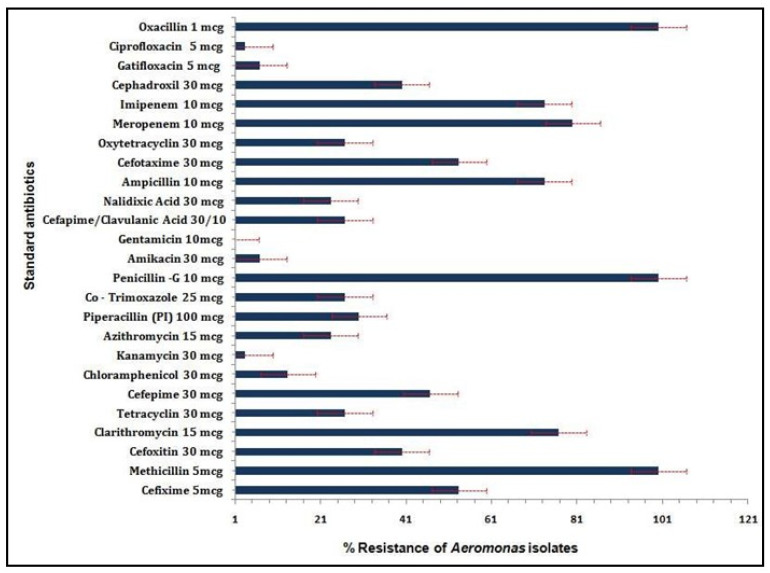
The antibiogram of *Aeromonas* strains (*n* = 30) representing the percent resistance against twenty-five commercial antibiotics.

Strains were sensitive to gentamicin but 100% resistant against β-lactam antibiotics methicillin, Penicillin-G, and oxacillin. Strains showed lowest resistance (30%) against piperacillin among the β-lactams. The strains showed least resistance against aminoglycosides ranging between 0–6.6%. All the strains with a MAR index of >0.2 were found to be multidrug resistant. Nearly 46.6% strains were found resistant to fourth generation cephalosporins (Figure 4 and Figure S2).

### 3.4. Association between Virulence Genes and Antibiotic Resistance

**Figure 5:**
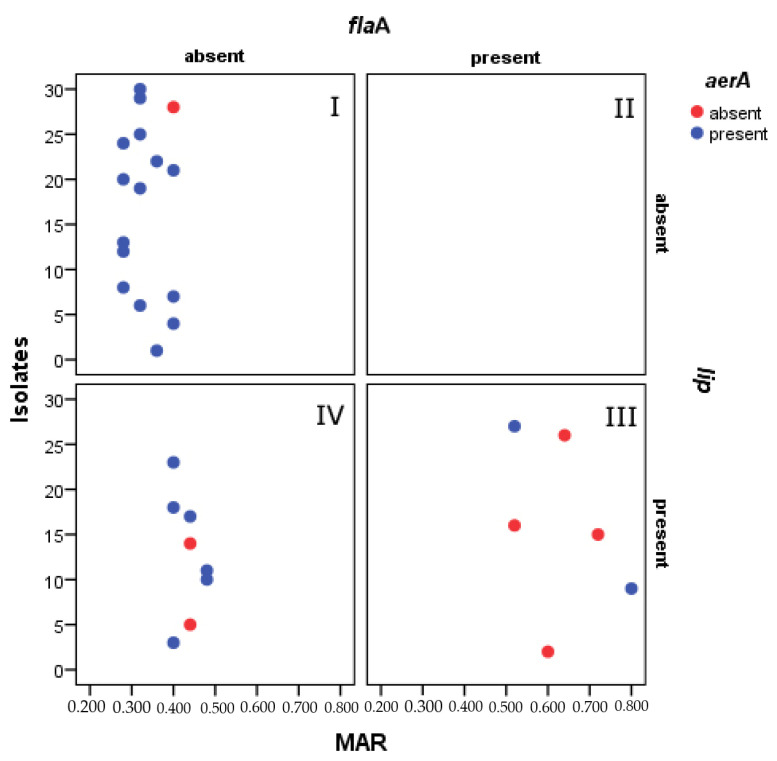
Scatter plot analysis of the distribution of *Aeromonas* strains (*n* = 30) based on multi-drug resistance index (MAR) and the presence of the tested virulence genes (*aer*A, *lip*, and *fla*A).

Association between the MAR and the virulence genes: *aer*A, *lip*, and *fla*A were confirmed to be statistically significant based on Pearson’s chi-square test. Chi-square values were as follows: [*X*2(10)] = 18.687 for MAR and *aer*A, *X*2(10) = 23.112 for MAR and *lip*, and *X*2(10) = 30.0 for MAR and *fla*A. Goodman and strength of association between variables were strong with a Cramer’s V value of >0.4. Scatter plot of strains (*n* = 30) vs. MAR value showed presence and absence of genes (Figure 5). Pictograph for the representative 30 isolates showed that the highest MAR index was found in strains carrying all three virulent genes. Additionally, out of 134 isolates, 61.94 % isolates were multiple antibiotic resistant, 8.20 % isolates were found be sensitive to all the tested antibiotics. The co-existence of virulence genes for aerolysin and lipase played a key role in multiple antibiotic resistance.

### 3.5. LD50 Determination

**Figure 6:**
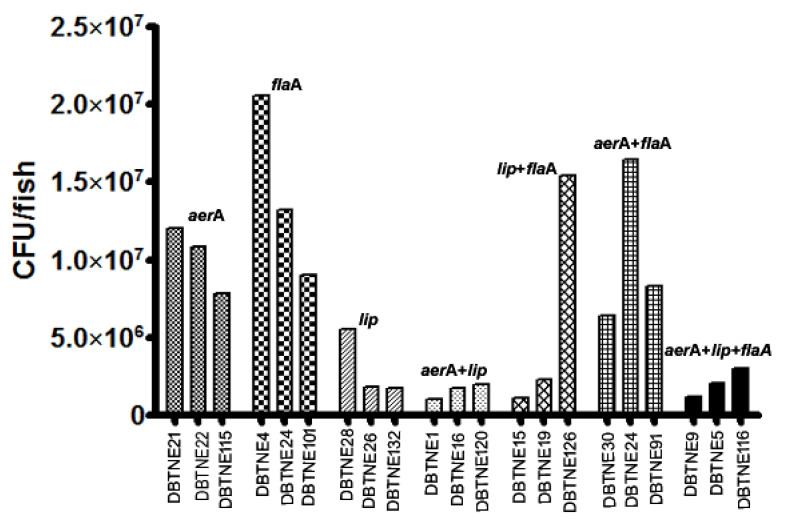
Graphical representation of LD50 values of *Aeromonas* isolates having virulence genes singularly and in combinations.

The average LD50 values for the isolates containing all the genes were 2.1 × 10^6^ CFU/fish. However, the lethal dose (50) for the isolates containing both the aerolysin and lipase genes was lower with an average of 1.6 × 10^6^ CFU/fish. The highest dose corresponded to isolates containing flagellin gene with an average LD50 1.42 × 10^7^ CFU/fish. Among the isolates, DBTNE9 (MZ723498) had the lowest LD50 1.05 × 10^6^ CFU/fish. Isolates expressing the lipase protein was found to be more virulent than aerolysin and flagellin (Figure 6).

### 3.6. Protein Network Analysis of Virulence Genes

#### 3.6.1. Aerolysin

**Figure 7:**
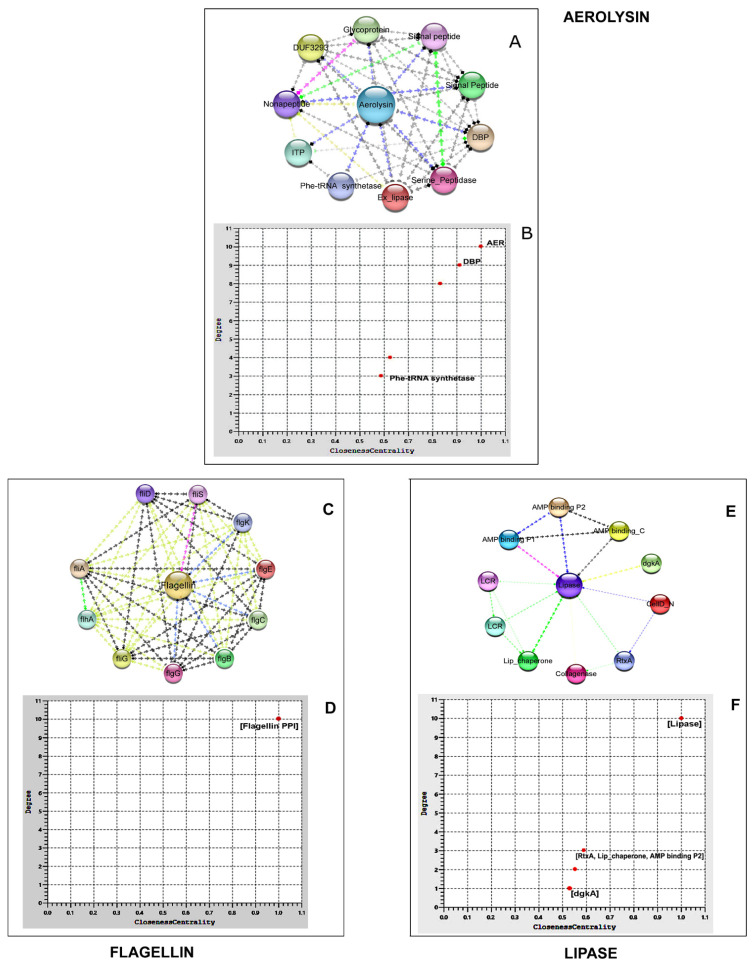
AEROLYSIN: (**A**): Protein-protein network analysis using STRING 11.0; (**B**) Scatter plot of the Degree centrality vs. Closeness centrality of the proteins in the aerolysin network. The nodes (*n* = 11) are represented in coloured balls and the edges are in arrow lines; blue: co-occurrence; pink: experimentally determined; green: neighbourhood; yellow: text mining; and black: co-expression. FLAGELLIN:(**C**) Protein–protein network analysis of flagellin from *A. hydrophila* ATCC7966 using STRING 11.0; (**D**) Scatter plot of the Degree centrality vs. Closeness centrality of the proteins in the flagellin network. The nodes (*n* = 11) are represented in coloured balls and the edges are in arrow lines; blue: co-occurrence; pink: experimentally determined; green: neighbourhood; yellow: text mining; and black: co-expression. LIPASE: (**E**) Protein–protein network analysis of lipase from A. hydrophila ATCC7966 using STRING 11.0; (**F**) Scatter plot of the Degree centrality vs. Closeness centrality of the proteins in the lipase network. The nodes (*n* = 11) are represented in coloured balls and the edges are in arrow lines; blue co-occurrence; pink: experimentally determined; green: neighbourhood; yellow: text mining; and black: co-expression.

The PPI shows 11 different proteins interacting with aerolysin in 41 different interacting pathways. It showed the strongest interaction with DNA binding proteins (DBP), prolyl tRNA synthetases with a Stringdb score of 0.719. The second Stringdb score belonged to the interaction with peptidase M4 of the thermolysin family (0.712). The PPI enrichment was confirmed to be significant (Table 2 and Table S2). Average local clustering coefficient of the interaction is 0.872, which ensured a highly connected network. Ten co-occurring genes had been detected, of which two were co-expressing genes. Degree centrality (importance of the node) vs. Closeness centrality (effect of the node in the overall interaction) scatter plot revealed that DBP prolyl-tRNA synthetases were important and influencing proteins and phenylalanyl-tRNA synthetase β-subunit (Phe-tRNA synthetase) had the least influence on the aerolysin protein network (Figure 7).

#### 3.6.2. Flagellin

The *fli*D flagellar hook-associated protein (Stringdb score: 0.975) yielded strongest interaction with flagellin. RNA polymerase sigma factor: *fli*A scored the second-best interaction (0.963). All 11 genes in the network were co-occurring and co-expressing. The PPI enrichment was significant with a clustering coefficient value of 1 (Table 2 and Table S3). The degree centrality vs. closeness centrality scatter plot showed that all of the 10 proteins exerted a uniform influence on *Aeromonas* strain flagellin network (Figure 7).

#### 3.6.3. Lipase

PPI for lipase revealed the highest interaction level (Stringdb score: 0.929) with its co-expressing gene, chaperon protein (Lip_chaperone). The second best interaction (0.907) was found with diacylglycerol kinase (dgkA) (0.907). Two co-occurring genes and two co-expressing genes were described along with *lip* and three neighbourhood genes. The PPI enrichment was significant with a clustering coefficient of 0.895 (Table 2 and Table S4). The degree centrality vs. closeness centrality scatter plot showed that AMP binding proteins, Lip_chaperone, and RtxA exerted more influence in the lipase protein network analysis (Figure 7).

## 4. Discussion

The farming of freshwater fish species has recently become intensive, with high stocking density resulting in high organic matter load and favourable conditions for bacterial disease outbreaks [45]. Disease caused by *Aeromonas* spp. is a result of triad interactions between the bacteria, fish, and aquatic environment. To better understand the pathogenesis details of the disease caused by *Aeromonas* spp. and their resistance to commonly used antibiotics, a comparative analysis of the virulence factors and the antibiogram of the bacteria were studied together. In the present study, the highest number was recorded in *A. veronii* (53.7%), followed by *A. hydrophila* (40.2%). The result is similar with the findings of Sun et al. (2016) [46], Foysal et al. [47], and Li et al. [48]. In contrast, *Aeromonas* isolates from other habitats and contaminated food were mostly from *A. caviae*, followed by *A. dhakensis* [49]. *A. veronii*, *A. hydrophila*, and *A. caviae* are the major aeromonads causing MAS in the freshwater aquaculture system of Assam, India [11]. Pathogens also cause hemorrhagic septicemia in fish, such as tilapia, carp, striped catfish, and long-whiskered catfish [50]. Result indicates that most *A. veronii* and *A. hydrophila* strains display high potential of pathogenicity in fish [51]. Ninety percent of *Aeromonas* strains were carrying significant virulence genes. However, 6.6% of the *Aeromonas* strains mostly isolated from *S. sarana*, carried none of the tested virulence genes. However, there are scanty reports of an *Aeromonas* infection in *S. sarana*. This could potentially be the reason why the bacteria failed to cause extreme septicemic manifestations in the particular fish host (Figure 1A). Hence, other virulence factors might be involved in the pathogenesis process.

In the present investigation, 76.6% of the strains carried the *aer*A gene, which was most prevalent among all the 30 species with the highest occurrence in *A. veronii* (52.17%). Gene codes for aerolysin, a pore-forming toxin causing hemolysis and soft tissue necrosis in the intestine of host [52]. High prevalence of aerolysin in the pathogenic strains of *Aeromonas* spp. has been also reported earlier [53,54]. Extracellular lipase (*lip*) is another virulence factor present in a broad range of *Aeromonas* spp. Gene alters the membrane permeability of the host tissues and facilitates bacterial colonization and proliferation [55]. In this study, 46.6% of the strains carried the *lip* gene, of which 42.85% were *A. hydrophila* strains from tilapia and carp. There are earlier studies on the prevalence of *lip* gene in 23.1% of *A. hydrophila* isolates of Nile tilapia [56]. Flagellar presence is considered a virulent determinant and vital for the adherence to the host tissue surface [57]. In the present study, the *fla*A gene encoding flagellin was harboured by 26.6% of the strains, of which 10% of each was *A. veronii* and *A. hydrophila*, and 6.6% constituted of *A. caviae*. Polar flagellum of the *Aeromonas* strains consists of two tandem subunits *fla*A and *fla*B, which polymerize to form the filaments [58].

Tested virulence genes *aerA*, *lip*, and *fla*A with their encoded proteins interact with several other proteins involved in the signaling pathways leading to pathogenesis [50]. In this study, aerolysin from *A. veronii* interacted strongly with DBP prolyl-tRNA synthetase (0.71) and its smaller paralog, YbaK. Prolyl-tRNA synthetase belongs to the ubiquitously expressed enzyme family of aminoacyl-tRNA synthetases (aaRS), performing a crucial role in protein biosynthesis, cell growth, and viability. Enzyme of bacterial origin is different from the eukaryotic one and widely studied as a promising antimicrobial drug target [59]. Second strongest interaction with aerolysin was that of M4 (MEROPS) protease of the zinc metallopeptidase family. It degrades extracellular proteins and peptides during bacterial growth [60]. Peptidases with elastolytic activity remove the C-terminal peptide of pro-aerolysin to convert it into virulent aerolysin [61]. It is a key pathogenetic factor of *Vibrio cholera*, *Enterococcus faecalis*, *Pseudomonas aeruginosa*, and *Clostridium perfringens*. Enzyme also provides stability to microorganisms to thrive under extremely adverse growth conditions. High-affinity inhibitors of the M4 protease family opened new therapeutic ways in clinical practice and computational biochemistry [62,63]. Nonapeptide, present in the aerolysin network, helps the toxin to depolarize the lipid bilayer permeability of the host cells with its calcium-binding domain and supports pore formation [64]. In our studied network, aerolysin and lipase were co-expressing. Similar correlation in the prevalence of both the genes was observed in *A.hydrophila* from tilapia [56].

Lipase shows its highest interaction with lipase chaperon (0.929) that helps in its proper folding and release through the periplasm in its active protease-resistant form [65]. Kinase and transferases activity of diacylglycerol kinase (dgkA) (0.90) present in the lipase network fulfills a specific task in bacterial membrane phospholipid biosynthesis, assisting in biofilm formation on the host surface [66]. It also interacts with the collagenase of the fish host tissues to breakdown the collagen barrier in the skin, bone, and scales [67]. Diacylglycerol kinase is an emerging human drug target for cancer, epilepsy, auto immunity, cardiac hypertrophy, hypertension, and Type II diabetes [68]. Cytotoxic RtxA protein in the lipase network plays a role in the adherence to the host membranes by actin filament reorganization, thereby facilitating all the molecular trafficking of the Gram-negative bacteria during infection. These proteins can be inferred to cumulatively assist host cell necrosis and apoptosis [69]. The polar flagellum region of *Aeromonas* strains interacts with 16 genes based in previous studies [70], of which 10 major genes have been included in the present interaction study. Functionally, proteins can be grouped as those encoding the flagellum hook (*flg*E, *flg*K, *fli*S, and *fli*D), rod (*flg*B, *flg*C, and *flg*G), biosynthesis (*flh*A), motor switch (*fli*G), and σ28factor (*fli*A). FliD protein with the strongest interaction (0.97) forms the basal part of the flagella regulating the mucin-specific adhesion of the bacteria on the host surface [71]. The σ28transcription factor (*fliA*) is an integral part of the flagellin gene network encoding the *Aeromonas* spp. polar flagellum. The *fli*A mutation leads to polar flagellar dysfunction and biofilm formation failure by *Aeromonas* spp. The *fli*A gene is responsible for regulating flagellin adherence and biofilm formation potential.

The pathogenicity of the isolates was found to be a direct measurement to virulence and toxicity. The co-existence of aerolysin and lipase was observed to be more lethal to fish. The presence of flagellin was comparatively avirulent. The study is in agreement with a previous study that used a mouse model challenged orally with *Aeromonas* [72]. Antibiogram was consistent with previous data explaining the intrinsic resistance of *Aeromonas* spp. to beta-lactam antibiotics [51]. Most strains were 100% resistant to β-lactam antibiotics except for piperacillin (30%), imipenem (73.3%), and meropenem (80%). We observed that 53.3% of the strains (31.2% *A. veronii* and 22.1% *A. hydrophila*) were showing the highest resistance to third generation cephalosporins (cefixime (56.6%) and cefotaxime (53.3%)) followed by fourth generation cefepime (46.6%). This phenomenon may be attributed to the presence of Class C cephalosporinases [73]. These strains were least resistant to first and second generation cephalosporins, which are contrary to most of the earlier studies. Similar resistance pattern was, however, observed in multidrug-resistant *Escherichia coli* isolated from German ICUs [74]. Moreover, 26.6% of the *A. hydrophila* strains exhibited low resistance against the combination of cefepime and clavulanic acid (CFC) due to the synergistic effect of both the antibiotics. Matter of fact, 82.35% of the cephalosporin-resistant strains contained at least one of the tested virulence genes. Our studies showed that all *Aeromonas* strains were susceptible to aminoglycosides except for *A. dhakensis* strain 21 and *A. veronii* strain 22 containing all the three virulence genes. These observations predict the correlation of antimicrobial resistance and presence of virulence factors. Earlier studies also reported the susceptibility of *Aeromonas* strains cultured in vitro and those obtained from natural water bodies to aminoglycoside antibiotics [25]. This means that the strain expressing the tested virulence genes also regulates the aminoglycoside modifying genes [33] while the sensitive strains are inhibited by protein synthesis blocking and cell membrane disruption [75]. All the *Aeromonas* strains were sensitive to quinolones and fluoroquinolones except for *A. caviae.* This phenomenon could be explained based on Type II topoisomerase quinolone resistance-determining regions of *A. caviae*. All the tested *Aeromonas* strains exhibited multidrug resistance with a MAR index of >0.2, which might be due to the presence of different virulence genes, antibiotic resistance genes, or exposure to antimicrobials in the environment [76]. MAR index was the highest (0.8) for *A. veronii* strain 6, which also carried all the tested virulence genes. In a recent study, 36% isolates of *Aeromonas* strains isolated from diseased rainbow trout were found to be multi-antibiotic resistant [77]. In another study, it was found that across a period of ten years, the fish-isolate *Aeromonas* spp. showed increased resistance to carbapenem, aztreonam, and third generation and fourth generation cephalosporins [78].

## 5. Conclusions

The divergence in the virulence profiles and antibiotic susceptibility patterns in different water bodies of Assam highlights the ability of *Aeromonas* spp. to adapt to the changing environmental conditions. The aerolysin gene is the most frequently distributed among the tested *Aeromonas* strains followed by flagellin and lipase gene. All the strains with at least one virulence gene were multidrug-resistant. This suggests a positive correlation between the presence of the virulence genes and antibiotic resistance profile in the *Aeromonas* strains of diseased fish origin. Cephalosporins (fourth generation) and aminoglycosides were comparatively effective against the *Aeromonas* strains. The cefepime and clavulanic acid combination (CFC) also showed inhibitory results against the tested strains. The six strains (1, 2, 4, 10, 16, and 17) isolated from aquaculture farms were mainly *A. veronii*, which had comparatively less resistance profile than wetland strains. The probability of using potential antibiotics in studied wetlands is very less. Therefore, the possibility of antibiotic leaching from other sources as inappropriate disposal of medical wastes cannot be ruled out. The findings of this study are important for the surveillance of pathogenic-resistant *Aeromonas* strains, which could potentially threaten human and animal health. This study also shows that there is an antibiotic resistance pattern in the wetland systems which needs to be further investigated.

## Figures and Tables

**Table 1:** Biochemical identification of representative *Aeromonas* isolates sequenced for 16S rRNA gene.

Sl No.	Biochemical Parameters	A 1	A 2	A 3	A 4	A 5	A 6	A 7	A 8	A 9	A 10	A 12	A 13	A 14	A 15	A 16	A 17	A 18	A 19	A 20	A 21	A 22	A 23	A 24	A 26	A 28	A 29	A 30	A 89	A 91	A 94
1	ONPG	+	+	+	+	+	+	+	+	+	+	+	+	+	+	+	+	+	+	+	+	+	+	+	+	+	+	+	+	+	+
2	Urease	−	−	−	−	+	−	−	−	−	−	−	−	+	−	−	−	−	−	−	−	−	−	−	−	−	−	−	+	−	−
3	Lysine utilization	+	+	+	+	−	+	+	+	+	+	+	+	−	+	−	+	+	+	+	+	+	+	+	+	+	+	+	−	+	+
4	Nitrate reduction	+	+	+	+	+	+	+	+	+	+	+	+	+	+	+	+	+	+	+	+	+	+	+	+	+	+	+	+	+	+
5	Ornithine utilization	+	+	−	+	−	−	−	−	−	−	−	−	−	−	−	−	−	−	−	−	−	+	−	−	−	−	−	−	−	−
6	Malonate utilization	−	−	−	−	−	−	−	−	−	−	−	−	−	−	−	−	−	−	−	−	−	−	−	−	−	−	−	−	−	−
7	VP	+	+	+	+	−	+	+	+	+	+	+	+	−	+	+	+	+	+	+	+	+	+	+	+	+	+	+	−	+	+
8	Esculin hydrolysis	+	−	+	+	+	+	+	+	+	+	+	+	+	+	+	+	+	+	+	+	+	−	+	+	+	+	+	+	+	+
9	Phenylalanine deamination	+	+	+	+	+	+	+	+	+	+	+	+	+	+	+	+	+	+	+	+	+	+	+	+	+	+	+	+	+	+
10	Citrate utilization	+	+	+	+	+	+	+	+	+	+	+	+	+	+	+	+	+	+	+	+	+	+	+	+	+	+	+	+	+	+
11	H_2_S production	−	−	−	−	−	−	+	−	−	+	+	−	−	+	−	+	+	−	+	−	+	−	+	+	−	−	+	−	−	+
12	Indole	+	+	+	+	+	+	+	+	+	+	+	+	+	+	+	+	+	+	+	+	+	+	+	+	+	+	+	+	+	+
13	Methyl red	−	−	−	−	−	−	−	−	−	−	−	−	−	−	−	−	−	−	−	−	−	−	−	−	−	−	−	−	−	−
14	Oxidase	+	+	+	+	+	+	+	+	+	+	+	+	+	+	+	+	+	+	+	+	+	+	+	+	+	+	+	+	+	+
15	Arabinose	+	+	+	+	−	+	+	+	+	+	+	+	−	+	+	+	+	+	+	+	+	+	+	+	+	+	+	−	+	+
16	Adonitol	−	−	−	−	−	−	−	−	−	−	−	−	−	−	−	−	−	−	−	−	−	−	−	−	−	−	−	−	−	−
17	Xylose	−	−	−	−	−	−	−	−	−	−	−	−	−	−	−	−	−	−	−	−	−	−	−	−	−	−	−	−	−	−
18	Rhamnose	−	−	+	−	−	+	+	+	+	−	−	+	−	+	+	+	−	+	+	+	−	+	−	−	+	+	+	−	+	+
19	Melibiose	+	−	−	−	+	−	−	−	−	−	−	−	+	−	−	−	−	−	−	−	−	−	−	−	−	−	−	+	−	−
20	Cellobiose	+	+	−	+	+	−	−	−	−	−	−	−	+	+	−	−	−	−	−	−	−	+	−	+	−	−	−	+	−	−
21	Raffinose	+	+	−	−	+	−	−	−	−	−	−	−	+	−	−	−	−	−	−	−	−	−	−	−	−	−	−	+	−	−
22	Saccharose	+	+	+	+	+	+	+	+	+	+	+	+	+	+	+	+	+	+	+	+	+	+	+	+	+	+	+	+	+	+
23	Trehalose	+	+	+	+	+	+	+	+	+	+	+	+	+	+	+	+	+	+	+	+	+	+	+	+	+	+	+	+	+	+
24	Glucose	+	+	+	+	+	+	+	+	+	+	+	+	+	+	+	+	+	+	+	+	+	+	+	+	+	+	+	+	+	+
25	Lactose	−	−	+	−	+	+	+	−	+	+	−	+	−	+	+	+	+	+	+	+	+	−	+	−	+	+	+	+	+	+

**Table 2:** Summary statistics of aerolysin, flagellin, and lipase protein–protein interaction network.

Sl No.	Parameters	Aerolysin	Flagellin	Lipase
1	Number of nodes	11	11	11
2	Number of edges	41	55	18
3	Avg. Number of neighbours	7.455	10.0	3.273
4	Network diameter	2	1	2
5	Characteristic path length	1.255	1	1.67
6	Clustering coefficient	0.872	1	0.804
7	Network Density	0.745	1	0.327
8	Network heterogeneity	0.264	0	0.6780
9	Network centralization	0.311	0	0.822
PPI enrichment *p*-value		1.94 × 10^−12^	<1.0 × 10^−16^	0.0271

## Data Availability

The data that support the findings of this study are available on request from the corresponding author.

## Supplementary Materials

The following supporting information can be downloaded at: https://www.mdpi.com/article/10.3390/life12121979/s1, Figure S1: Yellow colonies of *Aeromonas* spp. grown in Ampicillin Dextrin Agar base supplemented with ampicillin and vancomycin; Figure S2: Antibiotic sensitivity assay of representative isolates of *Aeromonas* spp.; Figure S3: PCR amplification of representative samples of 16S rRNA & virulence genes. Table S1: Sequence of oligonucleotide primers used in the present study; Table S2: Interactions score for the *aer*A of *A. veronii* B565 with other proteins in the network; Table S3: Interactions score for the *fla*A of *Aeromonas hydrophila* ATCC7966 with other proteins in the network; Table S4: Interactions score for the lip of *Aeromonas hydrophila* ATCC7966 with other proteins in the network.
