# Supplementary material for: Co-Prevalence of Virulence and Pathogenic Potential in Multiple Antibiotic Resistant Aeromonas spp. from Diseased Fishes with In Silico Insight on the Virulent Protein Network
Source: Life (Basel). 2022 Nov 25;12(12):1979. doi: 10.3390/life12121979 (PMC9781969; doi:10.3390/life12121979)
Supplement: Supplementary file 1 [file life-12-01979-s001.zip › life-2010038-supplementary.pdf]

life

Supplementary Materials

Co-Prevalence of Virulence and Pathogenic Potential in Multiple Antibiotic Resistant *Aeromonas* spp. from Diseased Fishes with *In Silico* Insight on the Virulent Protein Network

Supplementary Tables

Table S1. Sequence of oligonucleotide primers used in the present study.

Sl No.	Gene	Primer sequence	Reference
1.	*Aeromonas* Genus specific 16S rRNA	**F**: CTACTTTTGCCGGCGAGCGG	Gordon *et al*., 2007
		**R**: TGATTCCCGAAGGCACTCCC	
3.	*aer*A	**F**: CCTATGGCCTGAGCGAGAAG	Soler *et al*., 2002
		**R**: CCAGTTCCAGTCCCACCACT	
4.	*lip*	**F**: CAYCTGGTKCCGCTCAAG	
		**R**: GTRCCGAACCAGTCGGAGAA	
5.	*fla*	**F**: TCCAACCGTYTGACCTC	Mohamed *et al*., 2010
		**R**: GMYTGGTTGCGRATGGT	

Table S2. Interactions score for the *aer*A of *A. veronii* B565 with other proteins in the network.

Sl No.	Annotation 1	Annotation 2	Description	Score
1.	Aerolysin	DBP	DNA binding protein, prolyl-tRNA synthetases	0.719
2.	Aerolysin	Nonapeptide	Hemolysin_N	0.643
3.	Aerolysin	ITP	Ion Transport Protein	0.625
4.	Aerolysin	Signal peptide	-	0.614
5.	Aerolysin	Serine Peptidase	-	0.712
6.	Aerolysin	Signal peptide	-	0.655
7.	Aerolysin	Glycoprotein	-	0.590
8.	Aerolysin	DUF 3293	-	0.568
9.	Aerolysin	Phe-tRNA synthetase	-	0.691
10	Aerolysin	Ex_lipase	Extracellular lipase	0.655

Table S3. Interactions score for the *fla*A of *Aeromonas hydrophila* ATCC7966 with other proteins in the network.

Sl No.	Annotation 1	Annotation 2	Description	Score
1.	Flagellin	FlgB	Flagellar basal body rod protein	0.872
2.	Flagellin	FlgC	Flagellar basal body rod protein	0.904
3.	Flagellin	FlgE	Flagellar hook protein	0.924
4.	Flagellin	FlgG	Flagellar basal body rod protein	0.884
5.	Flagellin	FlgK	Flagellar hook-associated protein	0.923
6.	Flagellin	FlhA	Flagellar biosynthesis protein	0.937
7.	Flagellin	FliA	RNA polymerase sigma factor	0.963
8.	Flagellin	FliD	Flagellar hook-associated protein 2	0.975
9.	Flagellin	FliG	Flagellar motor switch protein	0.872
10.	Flagellin	FliS	Flagellar protein	0.910

Table S4. Interactions score for the *lip* of *Aeromonas hydrophila* ATCC7966 with other proteins in the network.

Sl No.	Annotation 1	Annotation 2	Description	Score
1.	Lipase	Lip_chap	The proteobacterial lipase chaperone is a lipase helper protein which may be involved in the folding of extracellular lipase during its passage through the periplasm	0.929
2.	Lipase	LCR	Low complexity region	0.612
3.	Lipase	LCR	Low complexity region	0.467
4.	Lipase	Collagenase	Peptidase_M9	0.491
5.	Lipase	AMP-binding P1	Long-chain-fatty-acid--CoA ligase homolog	0.812
6.	Lipase	RtxA	Structural toxin protein RtxA	0.546
7.	Lipase	AMP-binding P2	Long-chain acyl-CoA synthetase	0.837
8.	Lipase	AMP-binding_C	Long-chain-fatty-acid--CoA ligase (This is a small domain that is found C terminal to PF00501. It has a central beta sheet core that is flanked by alpha helices.)	0.800
9.	Lipase	CelD_N	Endochitinase	0.504
10.	Lipase	dgkA_prokar	Diacylglycerol kinase (Recycling of diacylglycerol produced during the turnover of membrane phospholipid)	0.907

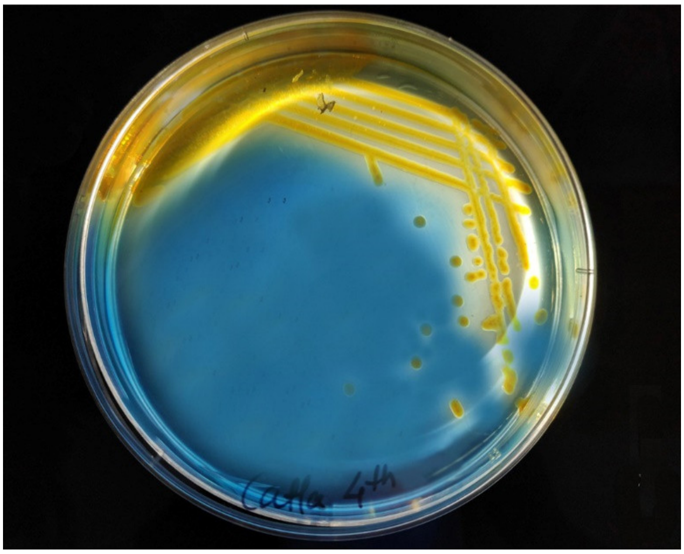

Figure S1. Yellow colonies of *Aeromonas* spp. grown in Ampicillin Dextrin Agar base supplemented with ampicillin and vancomycin.

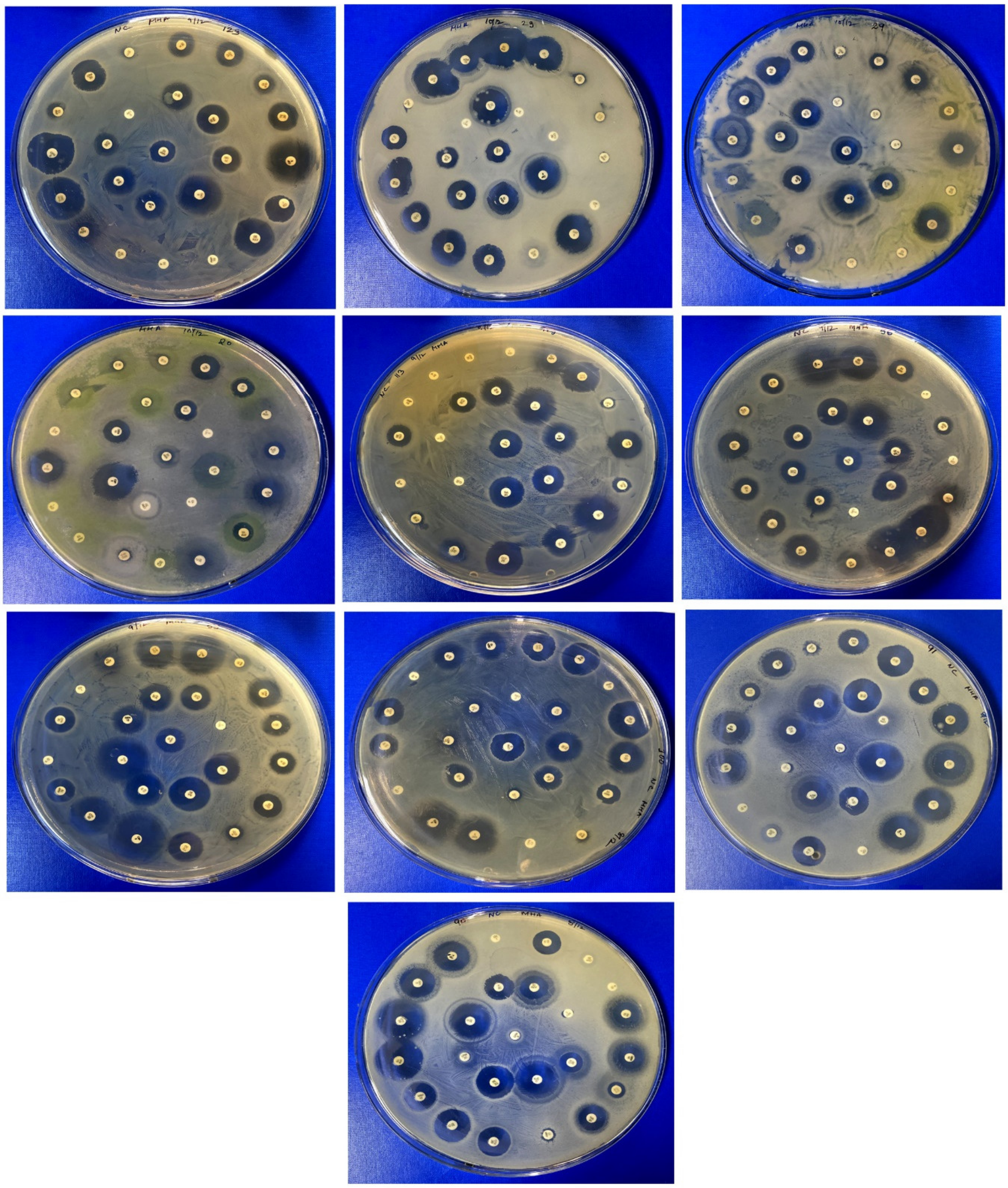

Figure S2. Antibiotic sensitivity assay of representative isolates of *Aeromonas* spp.

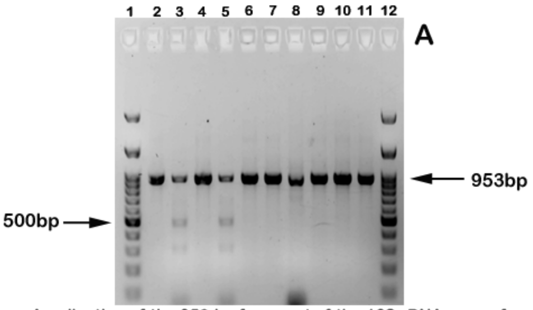

Amplication of the 953-bp fragment of the 16S rRNA gene of different species of *Aeromonas* isolates.
Lane 1&12 - 100-bp molecular weight marker;
lanes 2-11 - 953-bp region of the 16S rRNA gene PCR amplicon

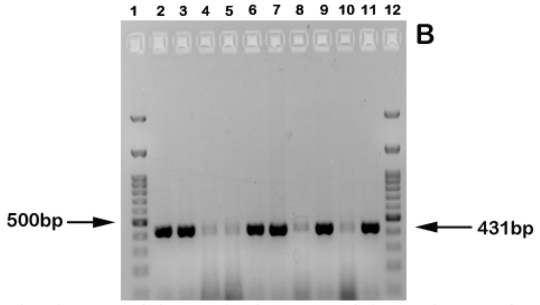

Amplication of the 431-bp fragment of the *aer*A gene of different species of *Aeromonas* isolates.
Lane 1&12 - 100-bp molecular weight marker;
lanes 2-11 - 431-bp region of the *aer*A gene PCR amplicon

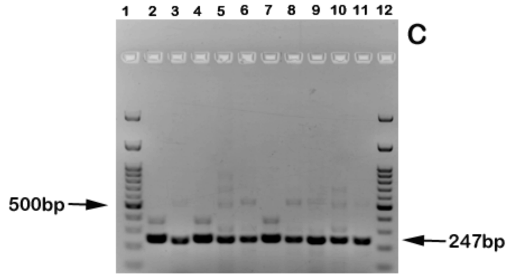

Amplication of the 247-bp fragment of the *lip* gene of different species of *Aeromonas* isolates.
Lane 1 & 12 - 100-bp molecular weight marker;
lanes 2-11 - 247-bp region of the *lip* gene PCR amplicon

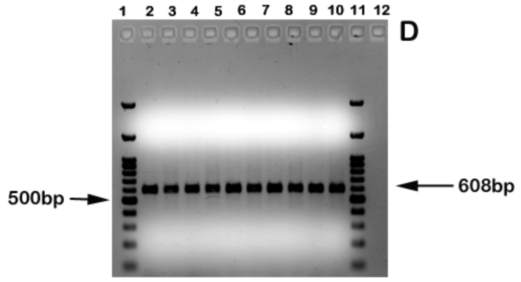

Amplication of the 608-bp fragment of the *flaA* gene of different species of *Aeromonas* isolates.
Lane 1 & 12 - 100-bp molecular weight marker;
lanes 2-11 - 608-bp region of the *flaA* gene PCR amplicon

Figure S3. PCR amplification of representative samples of 16S rRNA & virulence genes.
